# The role of basophils in acquired protective immunity to tick infestation

**DOI:** 10.1111/pim.12804

**Published:** 2020-11-13

**Authors:** Soichiro Yoshikawa, Kensuke Miyake, Atsunori Kamiya, Hajime Karasuyama

**Affiliations:** ^1^ Department of Cellular Physiology Okayama University Graduate School of Medicine, Dentistry and Pharmaceutical Sciences Okayama Japan; ^2^ Inflammation, Infection and Immunity Laboratory TMDU Advanced Research Institute Tokyo Medical and Dental University (TMDU) Tokyo Japan

**Keywords:** acquired immunity, basophil, histamine, IgE, IL‐3, mast cell, Tick

## Abstract

Ticks are blood‐feeding ectoparasites that transmit a variety of pathogens to host animals and humans, causing severe infectious diseases such as Lyme disease. In a certain combination of animal and tick species, tick infestation elicits acquired immunity against ticks in the host, which can reduce the ability of ticks to feed on blood and to transmit pathogens in the following tick infestations. Therefore, our understanding of the cellular and molecular mechanisms of acquired tick resistance (ATR) can advance the development of anti‐tick vaccines to prevent tick infestation and tick‐borne diseases. Basophils are a minor population of white blood cells circulating in the bloodstream and are rarely observed in peripheral tissues under steady‐state conditions. Basophils have been reported to accumulate at tick‐feeding sites during re‐infestation in cattle, rabbits, guinea pigs and mice. Selective ablation of basophils resulted in a loss of ATR in guinea pigs and mice, illuminating the essential role of basophils in the manifestation of ATR. In this review, we discuss the recent advance in the elucidation of the cellular and molecular mechanisms underlying basophil recruitment to the tick‐feeding site and basophil‐mediated ATR.

## 1 INTRODUCTION

Ticks (Acari: Ixodidae) are haematophagous and obligate ectoparasites that feed on blood of animals and humans. They are also major arthropod vectors that transmit a variety of tick‐borne pathogens, including *Borrelia burgdorferi* (the cause of Lyme disease), *Babesia microti* (the cause of babesiosis), and severe fever with thrombocytopenia syndrome virus.^1^, ^2^ While the application of acaricide is the most popular strategy for the prevention of tick infestation and tick‐borne diseases, repeated and prolonged use of such chemicals causes resistance against the acaricides in ticks and serious environmental pollution.^3^ To avoid such problems, other approaches are needed. It has been reported that acquired tick resistance (ATR) can be induced in some animal species after experiencing a single or multiple tick infestation(s),^4^, ^5^, ^6^ despite that ticks inject saliva substances, including immunosuppressive and anti‐inflammatory factors, into the host during blood feeding to facilitate their blood feeding.^7^, ^8^, ^9^, ^10^ ATR manifests itself as an increased duration of blood feeding and tick death, and reduction in blood meal volume, number of engorged ticks and egg production.^5^ Notably, animals with ATR show a reduced chance of the pathogen transmission when infested with pathogen‐bearing ticks.^11^, ^12^, ^13^, ^14^, ^15^ In humans, the frequency of Lyme disease is lower among individuals who express an immune reaction to *Ixodes scapularis* in comparison with those who express no such immune response.^16^ Moreover, some animal species with ATR show cross immunity to other strains of ticks, although this depends on the combination of animal and tick species.^4^ Therefore, a tick‐targeted vaccine is expected to reduce the number of patients with tick‐borne diseases by preventing pathogen transmission. To develop effective vaccines, it is important to investigate the cellular and molecular mechanisms of ATR.

ATR was first demonstrated by Trager in 1939.^4^ Single or repeated infestation of guinea pigs with larvae of the American dog tick, *Dermacentor variabilis*, led to the development of ATR, which prevented the subsequent attachment and engorging of larvae, starting within 2 weeks after the beginning of the 1st infestation and lasting for at least 3 months.^4^ This resistance was expressed systemically, rather than locally. For example, when the 1st infestation occurred in the left ear, ATR could be observed at the 2nd infestation site in flanks distant from the left ear. In some animals, ATR to infestation with larvae can be artificially produced via immunization of animals with larval antigens or can be transferred to naïve animals by means of the administration of serum or leukocytes isolated from tick‐resistant animals,^4^ suggesting that ATR is induced via an immunological reaction.

Basophils are a minor population among blood‐circulating leukocytes, which represent <0.5% of white blood cells.^17^, ^18^ They stay in the blood under steady‐state conditions and are recruited to peripheral tissues upon inflammation. Basophils share a number of morphological and functional similarities with mast cells, such as surface expression of the high‐affinity IgE receptor (FcεRI), the presence of basophilic granules in the cytoplasm, and the release of histamine and proteases stored in cytoplasmic granules.^17^, ^18^ Because of their rarity and similarity to mast cells, basophils had been sometimes thought as a subset of mast cells that circulate in the bloodstream and erroneously considered to share redundant roles with mast cells. Moreover, it was notoriously difficult to detect mouse basophils by using the standard method such as May‐Grunwald‐Giemsa staining because of fewer basophilic granules compared with basophils in other animal species, leading to the misunderstanding that mice lack basophils, and therefore basophils may not have any critical functions in other animal species as well. Recent advancement in the development of analytic tools, such as a basophil‐specific antibody and genetically engineered basophil‐deficient mice, revealed non‐redundant roles of basophils in various immune responses, including antiparasitic immune responses, allergic inflammation and autoimmune diseases.^19^, ^20^, ^21^ Thus, it is now appreciated that basophils are an indispensable cell type distinct from mast cells.

In this review, we discuss the cellular and molecular mechanisms underlying the acquired resistance to ticks observed in animal models of tick infestation, especially focusing on the role of basophils.

## 2 SKIN‐INFILTRATING BASOPHILS PLAY A CRITICAL ROLE IN ATR

Several animal species, including guinea pigs, rabbits, cattle and mice, show massive infiltration of immune cells at the tick re‐infestation sites.^5^ Interestingly, basophils, which rarely exist in the peripheral tissues under steady‐state conditions, were detected at the tick‐feeding site of some tick‐resistant animals.^22^ Although the frequency of basophils among cells accumulating at tick‐infested sites varied, depending on the combination of animal and tick species, more than 70% of infiltrating cells were basophils when guinea pigs were infested with *Dermacentor andersoni* larvae.^22^ Brown et al demonstrated the critical role of infiltrating basophils at the tick‐feeding sites in ATR by generating a rabbit serum against guinea pig basophils.^23^ Administration of the serum to guinea pigs before the 2nd infestation of *Amblyomma americanum* larvae resulted in the abolishment of the tick resistance, concomitantly with basophil ablation.^23^

In mice, denHollander et al^24^ reported that the BALB/c strain exhibited tick resistance to *D. variabilis* during the 3rd and subsequent infestations, but not the 2nd and the 1st infestations. While they failed to detect histopathologically the accumulation of basophils at tick‐feeding sites of tick‐resistant mice, numerous degranulated mast cells were observed at those sites.^24^ Matsuda et al demonstrated that mast cell‐deficient mice, WBB6F1‐*W/W^v^*, did not show ATR after re‐infestation with *Haemaphysalis longicornis* larvae, and that ATR was recovered by adoptive transfer of cultured mast cells.^25^, ^26^, ^27^, ^28^ As basophils were hardly detected at tick reinfection sites of mast cell‐sufficient WBB6F1‐^+/+^ mice even though the mice showed ATR,^25^, ^26^, ^27^, ^28^ it was hypothesized that mast cells rather than basophils play a critical role in the manifestation of ATR in mice, unlike in guinea pigs. However, it remained possible that the role of basophils in ATR might have been overlooked, because mouse basophils are hardly detected by means of histochemical staining, such as May‐Grunwald‐Giemsa staining, as mentioned earlier in this review. Steeves et al discovered the infiltration of basophils at the tick‐feeding site of mice re‐infested with *D. variabilis* larvae by using electron microscopy which can definitely identify mouse basophils.^29^ Mast cell‐deficient WBB6F1‐*W/W^v^* mice were able to develop acquired resistance to the infestation with larval *D. variabilis*, unlike the observation in mice infested with *H. longicornis*.^29^ Therefore, it remained unclear whether either mast cells or basophils or both are important for ATR in mice.

**FIGURE 1:**
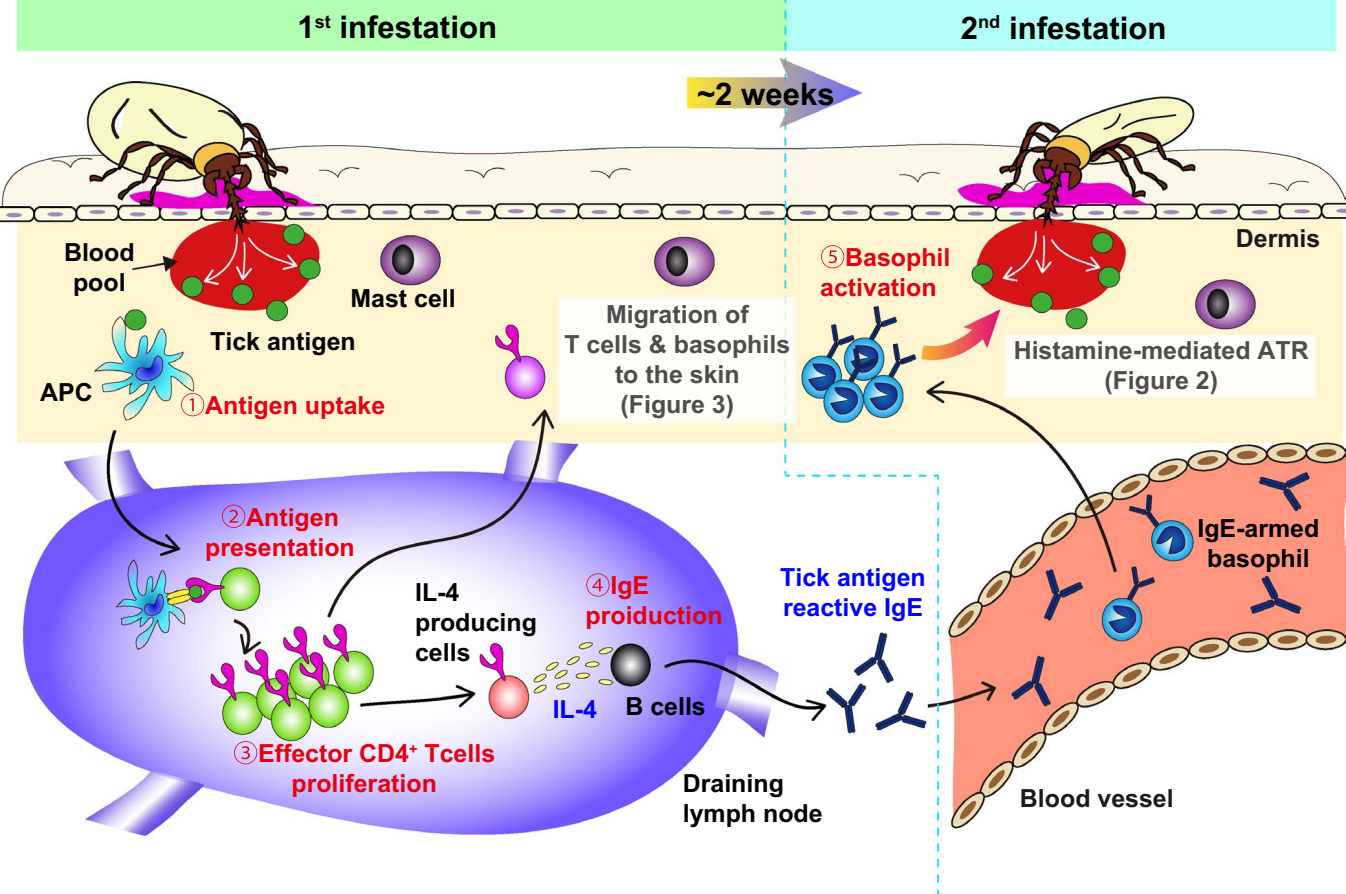
Tick antigen‐reactive IgE and its receptor FcεRI are essential for ATR. In the 1st tick infestation, (1) antigen‐presenting cells (APCs) capture tick antigens at tick‐feeding sites and then (2) migrate into the draining lymph node to present the antigens to naïve T cells. (3) Naïve CD4^+^T cells, which can respond to the tick antigens presented by APCs, proliferate and differentiate into effector CD4^+^T cells and (4) some of them become IL‐4 producing cells, such as Th2 or follicular helper T cells, to assist B cells to generate IgE reactive to the tick antigens. The tick antigen‐reactive IgE enters the bloodstream and binds to high‐affinity IgE receptor FcεRI on blood‐circulating basophils. During the 2nd infestation, IgE‐armed basophils infiltrate tick‐feeding sites and are activated by stimulation with IgE plus the tick saliva antigens, leading to the manifestation of ATR

**FIGURE 2:**
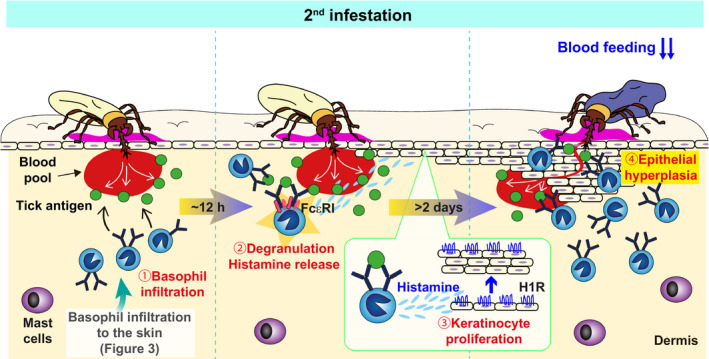
Histamine derived from basophils promotes the epidermal hyperplasia that leads to ATR. During the 2nd infestation, (1) IgE‐armed basophils infiltrate tick‐feeding sites and then (2) release histamine upon stimulation with IgE and tick antigens. (3) Histamine acts on keratinocytes and (4) promotes epidermal hyperplasia at tick‐feeding sites. Accordingly, larval ticks with short mouthparts cannot reach the blood pool, leading to tick detachment and reduction of blood feeding

Wada et al^30^ revisited the role of basophils and mast cells in the acquired resistance against larval *H. longicornis* in mice. ATR was defective in mast cell‐deficient mice, *Kit* ^W‐sh/W‐sh^, and was reconstituted by the adoptive transfer of cultured mast cells, consistent with Matsuda's report. Mouse mast cell protease‐8 (mMCP‐8) is specifically expressed in the granules of mouse basophils, but not mast cells, and the generation of a monoclonal antibody specific to mMCP‐8 has enabled the identification of mouse basophils in tissue sections.^31^ Histological examination using this antibody revealed the infiltration of basophils at tick‐feeding sites during the 2nd infestation, but not during the 1st infestation.^30^ To determine the role of basophils in ATR, those authors established a genetically engineered mouse model expressing the human diphtheria toxin receptor only on basophils.^30^ Because rodents are resistant to diphtheria toxin (DT) toxicity, basophils can be specifically depleted after DT treatment in this mouse model, while other types of cells, including mast cells, remain untouched. ATR was completely abolished by DT‐mediated basophil depletion just before the 2nd infestation,^30^ suggesting that not only mast cells, but also basophils, play a critical role in the ATR against *H. longicornis* larvae (Figures 1 and 2).

In humans, although the contribution of basophils to ATR remains unclear, infiltration of basophils was observed in skin lesions of ectoparasitic infestation, such as ticks and scabies.^32^, ^33^, ^34^ A patient with absence of basophils and eosinophils was reported to suffer from long‐term infestation of scabies.^35^ Thus, basophils might play an important role in ATR in humans.

In goats and guinea pigs, repeated infestation with nymphal *Amblyomma cajennense* or *Amblyomma triste* induced cutaneous basophilia at tick‐feeding sites of tick‐resistant animals.^36^, ^37^ This suggested that basophils might be involved in the manifestation of acquired resistance to nymphal ticks, similarly to larval ticks.

## 3 IMPORTANCE OF IgE RAISED AGAINST TICK SALIVA ANTIGENS AND ITS RECEPTOR FcεRI ON BASOPHILS FOR THE MANIFESTATION OF ATR

Several researchers have demonstrated that ATR was transferred to naïve animals by passive administration of sera from tick‐resistant animals, suggesting that tick antigen‐specific antibodies are important for the manifestation of ATR.^38^, ^39^, ^40^ The transfer of immune serum heated at 56°C for 2 hours, to inactivate IgE, failed to confer ATR on naïve mice, suggesting that IgE is a key factor for ATR.^28^ In fact, both B‐cell‐deficient (μMT) mice and IgE receptor FcεRI‐deficient (*Fcer1g* ^−/−^) mice showed impairment of the ATR to *H. longicornis* larvae.^30^ Although both basophils and mast cells are important for ATR, adoptive cell transfer revealed that the expression of the IgE receptor was required on basophils, but not on mast cells, for the occurrence of ATR.^30^ Therefore, the acquired resistance to larval *H. longicornis* in mice is mediated by the activation of basophils through FcεRI (Summarized in Figures 1 and 2).

Notably, in guinea pigs, adoptive transfer of sera from guinea pigs infested with larval *A. americanum* conferred ATR on naïve animals, regardless of whether the sera were heated or not to inactivate IgE.^41^ The administration of IgG_1_ purified from the infested animals produced ATR in naïve animals,^41^ suggesting that IgG_1_, rather than IgE, is responsible for ATR in guinea pigs.

## 4 HISTAMINE DERIVED FROM BASOPHILS, BUT NOT MAST CELLS, IS IMPORTANT FOR THE MANIFESTATION OF ATR

Cattle with resistance to *Boophilus microplus* showed an immediate hypersensitivity reaction after intradermal injection of crude tick antigens, which disappeared after the administration of anti‐histamine mepyramine maleate.^42^ Given the fact that the capacity for hypersensitivity is correlated with their resistance level in cattle,^43^ it can be assumed that ATR may possibly mediated by histamine. Indeed, in cattle and guinea pigs, the histamine content at tick‐feeding sites was higher in resistant animals than it was in naïve animals.^42^, ^44^ Moreover, treatment with anti‐histaminic agents abrogated ATR in tick‐resistant animals, including cattle and guinea pigs, while repeated subcutaneous injection of histamine induced the detachment of larval ticks when cattle, guinea pigs and rabbits were infested with *B. microplus*, *D. andersoni* and *Ixodes Ricinus*, respectively.^44^, ^45^, ^46^ These results suggest that histamine acts as an effector molecule in ATR. While several types of cells are known to release histamine, the cellular source of histamine involved in ATR remained unclear until recently.

Tabakawa et al demonstrated that histamine derived from basophils, but not mast cells, is important for the acquired resistance to *H. longicornis* larvae in mice (Figure 2).^47^ Those authors indicated that mice treated with an antagonist of the histamine H1 receptor (H1R), but not H2R, abolished ATR, while naïve mice injected with an agonist of H1R, but not H2R, H3R or H4R, exhibited ATR.^47^ Furthermore, H1R‐deficient mice showed abolishment of ATR, suggesting that H1R is essential for histamine‐mediated ATR. Both basophils and mast cells are essential for the manifestation of ATR in mice infested with *H. longicornis* larvae^30^ and are major producers of histamine among immune cells,^48^ suggesting that histamine derived from mast cells and/or basophils contributes to ATR. ATR was reconstituted in mast cell‐deficient mice by the adoptive transfer of mast cells isolated from mice whichever are sufficient or deficient for histamine decarboxylase (HDC) responsible for the generation of histamine.^49^ By contrast, the adoptive transfer of HDC‐sufficient, but not HDC‐deficient, basophils rescued ATR in basophil‐deficient mice, indicating that histamine derived from basophils, rather than mast cells, prevents *H. longicornis* larvae from blood feeding in mice. An intravital imaging analysis revealed that basophils mainly accumulated in the epidermis of tick re‐infested sites and surrounded the tick mouthparts, whereas most of mast cells were sparsely distributed in the dermis and located at distant sites from tick mouthparts.^47^ Because histamine is rapidly degraded by histamine‐degrading enzymes, histamine derived from basophils might affect tick feeding more effectively than that derived from mast cells.

How does histamine prevent ticks from feeding and attaching the host? One possibility is that itching and grooming can be elicited by histamine, leading to the facilitation of tick removal. In some experimental settings, tick infestation was carried out inside a plastic capsule attached to the skin, and hence ticks were protected from host grooming, suggesting that mechanisms other than grooming may also be operative in histamine‐mediated ATR. Tragers observed epidermal hyperplasia at *D. variabilis*‐feeding sites in tick‐resistant guinea pigs.^4^ Tabakawa et al^47^ also reported that epidermal hyperplasia was induced by re‐infestation with *H. longicornis* larvae or repeated subcutaneous injection of histamine. This hyperplasia was not induced in basophil‐depleted mice or HDC‐deficient mice.^47^ Keratinocytes express H1R and proliferate upon histamine treatment,^50^, ^51^, ^52^ suggesting that histamine released from activated basophils upon stimulation with IgE plus tick antigens acts on keratinocytes, leading to the promotion of epidermal hyperplasia, which inhibits tick blood feeding in the skin of resistant mice.

Intriguingly, Brown & Askenase and Bagnall demonstrated that treatment with anti‐histaminic agents did not alter the acquired resistance to *A. americanum* and *Ixodes holocyclus* in guinea pigs,^53^, ^54^ suggesting that these tick species are histamine‐resistant, in contrast to histamine‐sensitive tick species such as *H. longicornis*, *D. andersoni and B. microplus*. Of note, the histamine‐resistant ticks have long mouthparts (capitulum), whereas the histamine‐sensitive ticks possess short mouthparts.^55^, ^56^ The former ticks insert their mouthparts deep into the dermis of the host, where they create a blood pool. By contrast, the latter ticks establish a blood pool between the epidermis and dermis because their short mouthparts can only reach the epidermis.^57^ This may explain why the blood feeding of ticks with short mouthparts is strongly affected by the histamine‐induced epidermal hyperplasia, because their mouthparts are too short to reach the blood pool if the epithelial layer becomes thicker.

## 5 MOLECULAR MECHANISM OF BASOPHIL RECRUITMENT TO TICK‐FEEDING SITES

Cutaneous basophil hypersensitivity (CBH) was first discovered in guinea pigs and differs from the classical‐type (tuberculin‐type) delayed hypersensitivity.^58^ This hypersensitivity is characterized by subcutaneous basophilia at antigen‐injected sites in animals previously sensitized with the same antigen. T cells are responsible for the basophil infiltration in the lesions of CBH. It is believed that the basophil infiltration observed at tick‐feeding sites is a type of CBH reaction.^58^ However, the cellular and molecular mechanisms underlying basophil recruitment to tick‐feeding sites remained unclear.

**FIGURE 3:**
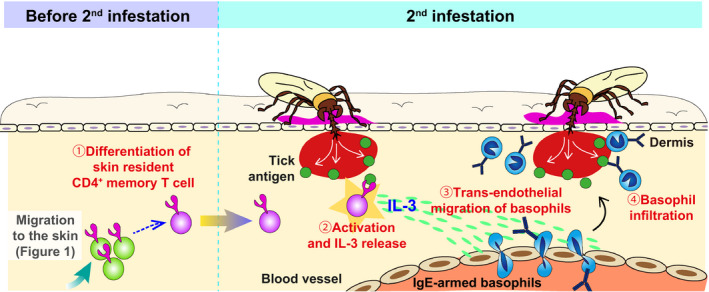
IL‐3 released from skin‐resident CD4^+^memory T cells is essential for basophil accumulation at tick‐feeding sites during re‐infestation. During the 1st tick infestation, tick antigen‐reactive CD4^+^T cells expand and migrate into the skin throughout the body, and (1) some of them remain in the skin as skin‐resident memory CD4^+^T cells. In the 2nd infestation, (2) such memory T cells stimulated with tick antigens produce IL‐3 that in turn (3) facilitates the trans‐endothelial migration of basophils, (4) leading to local cutaneous basophilia at tick‐feeding sites

Ohta et al addressed this issue by analysing mice re‐infested with *H. longicornis* larvae (Figure 3).^59^ Similar to CBH, the basophil accumulation at tick‐feeding sites during the re‐infestation was impaired in T‐cell‐deficient mice and was recovered by the adoptive transfer of memory CD4^+^ T cells isolated from tick‐resistant mice, demonstrating the importance of memory CD4^+^ T cells in basophil recruitment.^59^ The transcription of interleukin‐3 (*IL‐3*) gene was highly upregulated at tick‐feeding sites during the 2nd infestation, and mice deficient for this gene showed a lack of basophil infiltration.^59^ Furthermore, the transfer of IL‐3‐sufficient, but not ‐deficient, CD4^+^ T cells into T‐cell‐deficient mice led to basophil infiltration,^59^ indicating that IL‐3 derived from CD4^+^ T cells is essential for basophil accumulation at tick‐feeding sites. Of note, T cells capable of producing IL‐3 upon stimulation were detected in previously uninfested skin all over the body 14 days after the initiation of the 1st tick infestation and just before the 2nd infestation, and they exhibited a phenotype of tissue‐resident memory CD4^+^ T cells.^59^ Given that IL‐3 facilitates the adhesion of basophils to the endothelium,^60^, ^61^, ^62^ IL‐3 released from skin‐resident memory CD4^+^ T cells appears to induce the trans‐endothelial migration of basophils at tick‐feeding sites.

Taken together, one may assume the following steps towards the recruitment of basophils to tick‐reinfection sites (Figure 3). During the 1st tick infestation, tick antigen‐specific CD4^+^ T cells are activated in response to tick saliva antigens presented by antigen‐presenting cells migrating from the tick‐infested skin and expand in draining lymph nodes. Such CD4^+^ T cells migrate to the skin throughout the body and a fraction of them remain as skin‐resident, memory CD4^+^ T cells that are ready to respond to tick re‐infestation. When mice are re‐infested with ticks, the skin‐resident memory CD4^+^ T cells secrete IL‐3 in response to tick antigens, leading to basophil infiltration at the tick‐feeding sites.

In guinea pigs, complement deposition was found at tick‐feeding sites.^63^ Complement component‐3 (C3) and C5 exhibit potential chemotactic activity for basophils. The accumulation of basophils in the skin lesion of guinea pigs re‐infested with *D andersoni* larvae was abolished by treatment of animals with cobra venom which can deplete complement molecules. Importantly, basophil accumulation was normally observed in guinea pigs that are deficient for C4,^64^ suggesting that the alternative pathway, rather than the classical pathway, of complement activation might be important for basophil infiltration into tick‐bite sites in guinea pigs.

## 6 INHIBITORY EFFECT OF TICK SALIVA PROTEINS ON ATR

Ticks inject various saliva proteins, such as anticoagulation factors and anti‐inflammatory factors, into the host during blood feeding to facilitate their blood feeding.^7^, ^8^, ^9^, ^10^ In this section, we discuss the representative proteins that likely interfere with the basophil‐mediated ATR.

### 6.1 Histamine‐binding proteins

The histamine‐binding protein (HBP) was first identified as a tick lipocalin that has binding capacity for histamine.^65^, ^66^, ^67^ This group of proteins are produced in the salivary gland of some tick species and are injected into the host during blood feeding.^68^ They can outcompete the histamine receptors of the host and are believed to suppress the inflammation triggered by tick infestation. Although the histamine sensitivity of ticks may be correlated with the length of the tick mouthparts, as discussed above, the histamine‐mediated ATR may also be affected by the amounts HBPs injected by ticks.

### 6.2 IgG‐binding protein

IgG_1_ reactive to tick antigens is important for the manifestation of acquired resistance to *A. americanum* larvae in guinea pigs.^41^ The IgG‐binding protein (IGBP) discovered in *R. haemaphysaloides* can bind to IgGs of various animal species, such as rabbits and pigs. Moreover, the administration of anti‐IGBP serum resulted in the increased tick mortality and reduced blood feeding of engorged ticks,^69^ suggesting that IGBP is involved in the suppression of the host immune response. Wang and Nuttall found that IGBP‐MA, a member of the IGBP family, has a capacity of IgE binding,^70^ suggesting that IGBP‐MA may inhibit the IgE‐mediated ATR.

### 6.3 Anti‐complement proteins (ISAC, Salp20 and IRAC I/II)

Various species of ticks deliver anti‐complement factors, including ISAC, Salp20 and IRAC I/II, into the host during feeding, implying that blood feeding by ticks is affected by host complement molecules.^71^, ^72^, ^73^ These proteins block the alternative pathway of complement activation via the inactivation of C3 convertase. Because guinea pigs require this pathway to recruit basophils at tick‐feeding sites,^64^ anti‐complement proteins may prevent basophils from infiltrating tick‐feeding sites and therefore interfere with basophil‐mediated ATR.

## 7 CONCLUSION

Accumulating evidence suggests the following stepwise process in the development and manifestation of ATR (Figures 1, 2, 3). During the 1st infestation, antigen‐presenting cells (APCs), such as dendritic cells and Langerhans cells, capture tick saliva antigens at tick‐feeding sites and then migrate into the draining lymph node (Figure 1). Naïve CD4^+^ T cells, which can respond to tick antigens presented by APCs, proliferate and develop into type 2 helper T (Th2) cells and follicular helper T (Tfh) cells in the draining lymph node. They help B cells to produce tick antigen‐specific IgE antibodies that circulate in the bloodstream and bind to FcεRI on basophils. Some of tick antigen‐specific CD4^+^ effector T cells migrate into the skin throughout the body, and a fraction of them stay in the skin as skin‐resident CD4^+^ memory T cells. Upon re‐infestation of ticks, such skin‐resident CD4^+^ memory T cells secrete IL‐3, which in turn acts on endothelial cells close to tick‐feeding sites and facilitates the transmigration of blood‐circulating IgE‐armed basophils (Figure 3). Subsequently, skin‐infiltrating basophils release histamine upon stimulation with tick antigens and IgE/FcεRI on their surface. Histamine acts on keratinocytes and promotes epidermal hyperplasia at tick‐feeding sites that hampers tick attachment and tick feeding (Figure 2). Several issues still remain unanswered, including the role of mast cells in ATR. Further studies on the development and manifestation of ATR are needed to establish effective vaccines against ticks and tick‐borne diseases.

## CONFLICT OF INTEREST

The authors have no conflict of interest to disclose.

## AUTHOR CONTRIBUTIONS

SY and HK discussed and planned the review. SY wrote the first draft of the manuscript. SY, KM, AK and HK contributed to writing the final manuscript.

### Peer Review

The peer review history for this article is available at https://publons.com/publon/10.1111/pim.12804.
